# Clinical features, risk factors, diagnosis, and treatment of trimethoprim-sulfamethoxazole-induced hypoglycemia

**DOI:** 10.3389/fendo.2023.1059522

**Published:** 2023-02-08

**Authors:** Chunjiang Wang, Weijin Fang, Zuojun Li, Linli Sun

**Affiliations:** ^1^ Department of Pharmacy, The Third Xiangya Hospital, Central South University, Changsha, Hunan, China; ^2^ Department of General Surgery, The Third Xiangya Hospital, Central South University, Changsha, Hunan, China

**Keywords:** hypoglycemia, trimethoprim-sulfamethoxazole, pneumocystis pneumonia, seizure, neuroglycopenic symptoms

## Abstract

**Objective:**

Hypoglycemia is a sporadic and serious adverse reaction of trimethoprim-sulfamethoxazole (TMP-SMX) due to its sulfonylurea-like effect. This study explored the clinical characteristics, risk factors, treatment, and prognosis of TMP-SMX-induced hypoglycemia.

**Methods:**

Case reports and series of TMP-SMX-induced hypoglycemia were systematically searched using Chinese and English databases. Primary patient and clinical information were extracted for analysis.

**Results:**

A total of 34 patients were reported from 31 studies (16 males and 18 females). The patients had a median age of 64 years (range 0.4-91), and 75.8% had renal dysfunction. The median duration of a hypoglycemic episode was six days (range 1-20), and the median minimum glucose was 28.8 mg/dL (range 12-60). Thirty-two patients (97.0%) showed neuroglycopenic symptoms, with consciousness disturbance (30.3%) and seizure (24.2%), sweating (18.2%), confusion (15.2%), asthenia (12.1%) being the most common symptoms. Fifteen patients (44.1%) had elevated serum insulin levels, with a median of 31.8 μU/mL (range 3-115.3). C-peptide increased in 13 patients (38.2%), with a median of 7.7 ng/mL (range 2.2-20). Complete recovery from symptoms occurred in 88.2% of patients without sequelae. The duration of hypoglycemia symptoms was 8 hours to 47 days after the intervention. Interventions included discontinuation of TMP-SMX, intravenous glucose, glucagon, and octreotide.

**Conclusion:**

Hypoglycemia is a rare and serious adverse effect of TMP-SMX. Physicians should be aware of this potential adverse effect, especially in patients with renal insufficiency, increased drug doses, and malnutrition.

## Introduction

Trimethoprim-sulfamethoxazole (TMP-SMX), AKA co-trimoxazole, was approved in 1968 for treating urinary tract infections, uncomplicated sinusitis, and chronic bronchitis (1). Oral and intravenous preparations are manufactured from a fixed ratio of 1:5 of trimethoprim to sulfamethoxazole. TMP-SMX is also the therapy for treating Pneumocystis carinii pneumonia (PCP) (2).

The most common adverse reactions of TMP-SMX are rash, allergic reaction, gastrointestinal discomfort, hyperkalemia, nephrotoxicity, and pancytopenia (3). In rare cases, TMP-SMX can also cause severe hypoglycemia that is often overlooked, leading to fatal outcomes. Current knowledge about TMP-SMX-induced hypoglycemia is based primarily on case reports, and the specific clinical features are unclear. Here, we discuss the clinical features, risk factors, treatment, and prognosis of hypoglycemia induced by TMP-SMX to provide a basis for the rational use of TMP-SMX.

## Methods

### Search strategy and selection criteria

Case reports, case series, and clinical studies of cotrimoxazole-induced hypoglycemia were searched from Chinese and English databases, including Wanfang, China National Knowledge Infrastructure, China Science and Technology Journal Database, PubMed, OVID, Web of Science, Embase, and Cochrane Library. The search period was limited from January 1, 1968, to July 31, 2022. The searches were performed using subject and free words, including “trimethoprim-sulfamethoxazole” [MeSH] OR “trimethoprim” [MeSH] OR “sulfamethoxazole” [MeSH] OR “SMX” [MeSH] OR “TMP” [MeSH] OR “co-trimoxazole” [MeSH] AND “hypoglycemia” [MeSH] OR “blood glucose” [MeSH] OR “glycaemia” [MeSH]. There was no language restriction. Mechanistic studies, animal studies, reviews, and duplicate reports were excluded.

### Data extraction

The following data were extracted using self-designed tables: age, sex, underlying diseases, concomitant medications, indications, dosage regimens, risk factors, clinical symptoms and signs, laboratory tests (blood glucose, insulin, C-peptide, liver function, renal function), imaging studies, treatment, and prognosis.

### Diagnostic criteria for hypoglycemia

According to the latest diagnostic criteria for hypoglycemia of the American Diabetes Association, hypoglycemia can be diagnosed when the blood glucose level of diabetic patients is ≤70 mg/dL (≤3.9 mmol/L). In contrast, the blood sugar of non-diabetic patients is less than 55 mg/dL (3.0 mmol/L) (4).

### Statistical analyses

SPSS Statistics 22.0 (IBM, Armonk, NY, USA) was used for statistical analysis. Enumeration and measurement data were represented by n (%) and the median value (range, minimum and maximum values), respectively.

## Results

**Figure 1:**
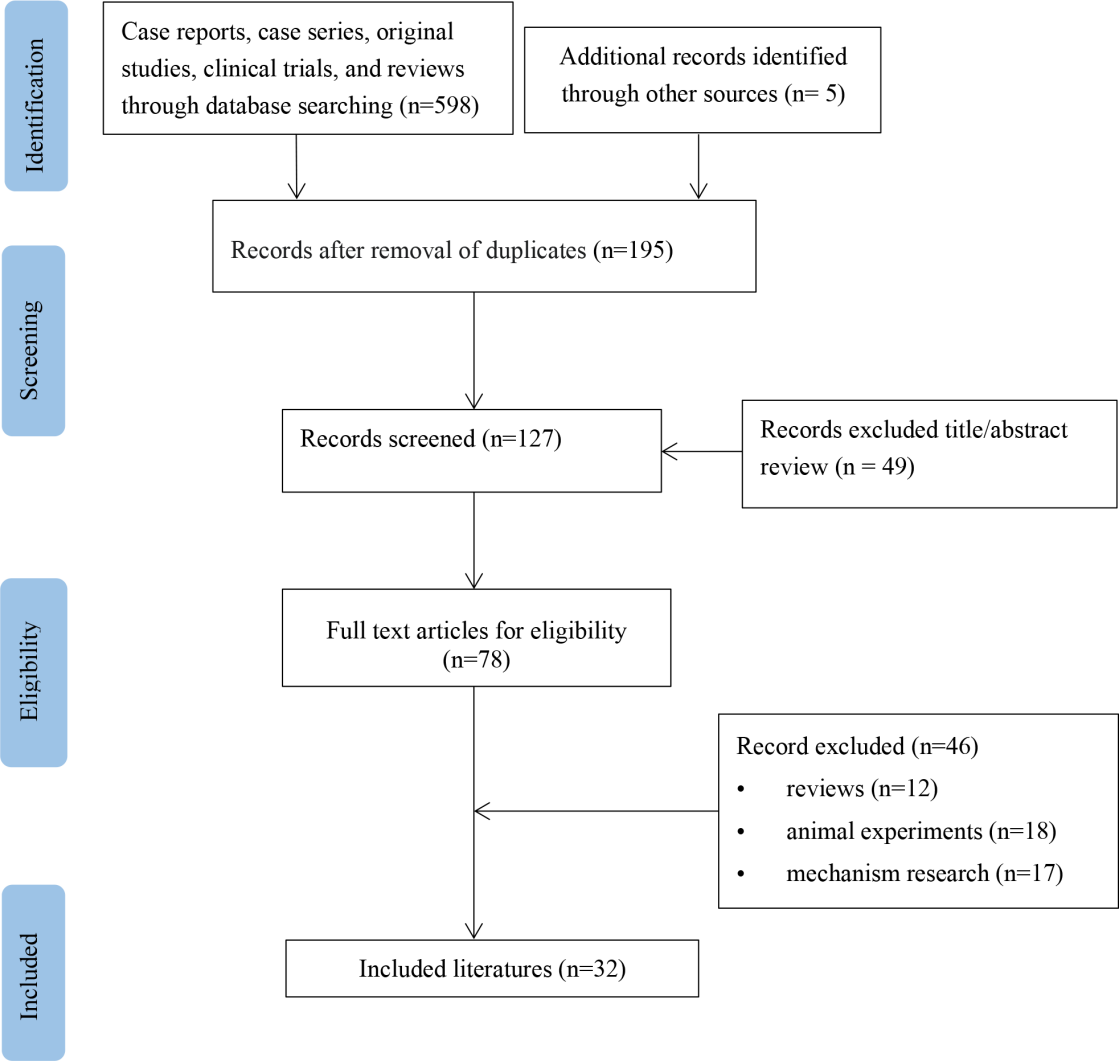
Flow chart of study selection process for reported cases of trimethoprim-sulfamethoxazole-induced hypoglycemia.

A flow diagram for the study is provided in **Figure 1**. According to the inclusion and exclusion criteria, 34 patients from 32 studies were included (**Table 1**) (5–36). The basic information about these patients is summarized in **Table 2**. These patients (16 men and 18 women) were mainly from North America (38.2%), Europe (44.1%), and Asia (7.6%), with a median age of 64 years (range 0.4-91). Medical history was available in 33 patients (97.1%), including 9 (27.3%) with type 2 diabetes and 2 (6.1%) with hepatitis. Ten patients (29.4%) had malnutrition. Twenty-two patients (43%) were taking concomitant drugs, including 13 (38.2%) taking drugs that could cause hypoglycemia, such as beta-adrenergic antagonists, quinolones, angiotensin-converting agent enzyme inhibitors (ACEI), propoxyphene, and hypoglycemic medications. The median daily dose of sulfamethoxazole is 3,200 mg (range 400-9,600). The median duration of TMP-SMX treatment before the hypoglycemia episode was six days (range 1-20).

**Table 1:** Summary of clinical information of 34 patients.

reference	age/sex	indication	daily dose (mg/d)	duration(d)	symptoms	serum glucose (mg/dL)	Insulin (μU/ml)	C-peptide (ng/mL)	serum creatinine (mg/dL)	Management
5	65/f	UTI	320/1600 po	10	seizure, dyspnea, lethargic	27	36	na	6.1	discontinued, dextrose iv
6	63/m	pyogenic arthritis	960/4800 po; 320/1600 po	5/na	seizures, mental status changes	26	58.7	na	HD	dose decreased
7	85/f	UTI	320/1600 po	7	confusion, loss of consciousness	24	3	na	1.3	discontinued, dextrose iv
7	74/f	UTI	320/1600 po	na	loss of consciousness	12	6	na	8.2	discontinued, dextrose iv
8	69/f	UTI	na	2	nausea, vomiting, weakness, slurred speech, numbness	48	na	na	normal	discontinued, dextrose iv
9	62/m	PCP	960/4800 po	5	consciousness disturbance	20	34.5	na	3.3	discontinued
10	64/m	PCP	1280/6400 iv	2	loss of consciousness	36	na	na	9.5	dextrose iv
11	34/m	PCP	na	6	stuporous	18	12	↑	normal	discontinued, dextrose iv
12	88/f	UTI	320/1600 po	4	GTCS, incoherence, confusion	33	na	NA	1.2	discontinued, dextrose iv
13	19/f	na	320/1600 po	1	confusion	17	na	NA	PD	dextrose iv
14	36/m	PCP	960/4800 iv, 1920/9600 po	8	tremor, loss of consciousness, seizure, sweating	28.8	na	4.3	3.6	discontinued, dextrose iv,diazepam
15	54/f	PCP	1280/6400	5	neuroglycopenic symptoms	36	24.2	7.7	normal	discontinued, dextrose iv
16	73/m	HAP	1280/6000 iv;600/3000 iv	6/9	asymptomatic	22	na	na	5.1	discontinued, dextrose iv, low-dose re-challenge
17	91/f	UTI	640/3200 po	7	decreased level of consciousness	34	na	na	1.7	discontinued
18	41/m	PCP	640/3200 po	6	tremor, sweating, disorienting, unresponsive	18	30.2	12.6	1.4	discontinued
19	5m/f	PCP	20/100 mg/kg per d	3	generalized convulsion	18	16.4	4.88	normal	dose decreased, diazoxide
20	46/m	PCP	1280/6400 po	18	GTCS, altered state of consciousness, falls	28.8	na	na	2	discontinued, dextrose iv
21	76/m	UTI	160/800	5	symptomatic hypoglycemia, inability to speak	34	na	na	2	discontinued, dextrose iv
22	44/f	PCP	1920/9600 iv	7	sweating, asthenia, dizziness	59	40	na	normal	continued, dextrose iv
22	24/m	PCP	960/4800 iv	20	sweating, asthenia, confusion, nausea, dizziness	56	80	na	normal	discontinued, dextrose iv
23	83/m	prophylaxis	160/800	na	loss of muscle tone, pale skin and mucous membranes	28	3.2	2.2	1.7	discontinued, dextrose iv
24	56/f	PCP	no	5	coma	30.6	2ULN	2ULN	CKD 5	discontinued, dextrose iv
25	52/f	prophylaxis	80/400	7	dizziness, hunger, headaches, sweating.	30.6	41.1	6.1	> 60 *	continued, oral sugar
26	60/f	UTI	160/800	3	tremor, sweating, fatigue	60	na	na	normal	discontinued
27	71/m	PCP	1120/5600	11	coma, acute neurological deterioration, nervous breakdown, hypothermia	24	na	na	3.2	discontinued, dextrose iv
28	69/m	PCP	960/4800	4	Tonic clonic seizure	28.8	95.9	12.8	AKI	discontinued, dextrose iv, glucagon
29	18/f	prophylaxis	80/400 po	2	na	43.2	8.1	11.7	0.5	discontinued, dextrose iv
30	85/m	UTI	320/1600 po	7	pale, altered state of consciousness	35	8.8	4.57	1.79	discontinued, dextrose iv
31	75/f	PCP	96 mg/kg/day	10	lost consciousness	20	na	na	53.7 *	discontinued, oral sugar, low-dose re-challenge
32	64/m	PCP	1280/6400 po	5	delirious, spoke nonsense words, displayed dancing arms, deliration	30.6	115.3	19.55	49.67*	dose decreased
33	73/m	bacteremia	160/800 2d, 960/4800 po	8	lethargy, visual hallucinations	45	31.8	4.5	1.3	Discontinued, hydrocortisone and dextrose iv, intramuscular glucagon and octreotide.
34	62/f	Cerebral toxoplasmosis	320/1600	6	GTCS	21	99	20	na	discontinued, dextrose iv, glucagon
35	64/f	PCP	no	na	confusion	21.6	no	15.5	AKI	discontinued, dextrose iv, glucagon
36	79/f	UTI	320/1600 po	6	consciousness disorder, coma, wandering, sweating	28.8	na	na	na	discontinued, dextrose iv

AKI, acute kidney injury; CKD, Chronic kidney disease; GTCS, generalized tonic clonic seizure; HD, hemodialysis; na, not applicable; PCP, Pneumocystis carinii pneumonia; PD, peritoneal dialysis; iv, intravenous; ULN, upper limit of normal value; UTI, urinary tract infection.

*Represents estimated glomerular filtration rate (mL/min).

AKI, acute kidney injury; CKD, Chronic kidney disease; GTCS, generalized tonic clonic seizure; HD, hemodialysis; na, not applicable; PCP, Pneumocystis carinii pneumonia; PD, peritoneal dialysis; iv, intravenous; ULN, upper limit of normal value; UTI, urinary tract infection.

*Represents estimated glomerular filtration rate.

**Table 2:** Summary of basic information of 34 patients.

Parameter		Value
Sex	F M	18 (52.3%) 16 (47.1%)
Age, years		64 (0.4,91) ^b^
Country	USA UK Canada Italy Japan, Turkey, France, China Portugal, Iran, Israel, Spain, Barbados	8 (23.5%) 6 (17.7%) 4 (11.8%) 3 (8.8%) 2 (5.9%) 1 (2.9%)
Onset time (days) (30) ^a^		6 (1,20) ^b^
Daily dose (mg) (29) ^a^		3200 (400,9600) ^b^
Indication (33)^a^	PCP UTI prophylaxis HAP, pyogenic arthritis, bacteremia cerebral toxoplasmosis	16 (48.5%) 10 (30.3%) 3 (9.1%) 1 (3.0%)
Medical history (33)^a^	type 2 diabetes autoimmune disease AIDS hypertension cardiovascular disease hematological tumor cancer nervous system disease, nephrolithiasis, osteoporosis, kidney transplant, COPD epilepsy, hypothyroidism	9 (27.3%) 8 (24.2%) 6 (18.2%) 4 (12.1%) 4 (12.1%) 3 (6.1%) 3 (6.1%) 2 (6.1%) 2 (6.1%) 1 (3.0%)
Combination therapy (22) ^a^	prednisone antibiotics hypoglycemic drugs diuretics propoxyphene H2 blockers antihypertensive drugs beta blocker Immunosuppressant antiviral drugs proton pump inhibitor	6 (27.3%) 5 (22.7%) 5 (22.7%) 5 (22.7%) 4 (18.2%) 4 (18.2%) 4 (18.2%) 3 (13.6%) 3 (13.6%) 3 (13.6%) 2 (9.1%)

PCP, Pneumocystis carinii pneumonia; UTI, urinary tract infection; COPD, chronic obstructive pulmonary disease; HAP, hospital acquired pneumonia; AIDS, acquired immune deficiency syndrome.

a Represents the number of patients out of 34 in whom information regarding this particular parameter was provided.

b Median (minimum-maximum).

### Clinical symptoms

Thirty-three patients had documented clinical symptoms, of which 32 (97.0%) developed neurological hypoglycemia symptoms and 1 (3.0%) had asymptomatic hypoglycemia. The most common symptoms during hypoglycemia episodes were consciousness disturbance (30.3%) and seizure (24.2%), followed by sweating (18.2%), confusion (15.2%), asthenia (12.1%), tremor (9.1%), dizziness (9.1%), coma (9.1%) and lethargic (9.1%). Other rare symptoms and signs include dyspnea, hypothermia, visual hallucinations, and numbness. Details are shown in **Table 3**.

**Table 3:** Summary of clinical symptoms and laboratory tests of 34 patients.

Parameter		Value
Symptoms and signs (33)^a^	asymptomatic neuroglycopenic symptoms	1 (3.0%) 32 (97.0%)
	consciousness disturbance seizure sweating confusion asthenia tremor dizziness coma lethargic pale skin and mucous membranes nausea deliration other rare symptoms: dyspnea, slurred speech, numbness, incoherence, inability to speak, disorienting, nonresponsive, fall, loss of muscle tone, hunger, hypothermia, dehydration, visual hallucinations, spoke nonsense words, displayed dancing arms, headaches	10 (30.3%) 8 (24.2%) 6 (18.2%) 5 (15.2%) 4 (12.1%) 3 (9.1%) 3 (9.1%) 3 (9.1%) 3 (9.1%) 2 (6.1%) 2 (6.1%) 2 (6.1%) 1 (3.0%)
Serum glucose (mg/dL)		28.8 (12,60) ^b^
Insulin (μU/mL) (19)^a^		31.8 (3,115.3) ^b^
	elevated normal	15 (78.9%) 4 (21.1%)
C-peptide (ng/mL) (13)^a^	elevated	13 (100%) 7.7(2.2,20) ^b^
Renal (33) ^a^	normal renal impairment*	8 (24.2%) 25 (75.8%)
Liver (22)^a^	hepatitis normal	2 (9.1%) 20 (90.1%)

* Renal impairment were categorized according to their estimated creatinine clearance at screening: normal renal function (≥ 90 mL/min/1.73 m^2^), mild impairment (60–89 mL/min/1.73 m^2^), moderate impairment (30–59 mL/min/1.73 m^2^) and severe impairment (15–29 mL/min/1.73 m^2^).

a Represents the number of patients out of 34 in whom information regarding this particular parameter was provided.

b Median (minimum-maximum).

### Laboratory test

The median lowest serum glucose measured was 28.8 mg/dL (range 12-60). Of the 19 patients measured, 15 (78.9%) had elevated serum insulin levels, with a median of 31.8 μU/mL (range 3-115.3). C-peptide levels increased in all 13 measured patients, with a median of 7.7 ng/mL (range 2.2-20). Renal impairment occurred in 25 of 33 patients (75.8%), and hepatitis occurred in 2 of 22 patients (9.1%). Details are shown in **Table 3**.

### Treatment and prognosis

TMP-SMX was immediately discontinued in 27 patients (79.4%), continued in 2 patients (5.9%), and the dose decreased in 3 patients (8.8%). One case (2.9%) did not describe whether treatment was discontinued or changed information. The management of TMP-SMX was not described in one patient. Thirty patients (88.2%) received intravenous glucose immediately, 2 (5.9%) received oral glucose, and 1 (2.9%) received carbohydrate supplementation. In addition, four patients (11.8%) received glucagon, and one each (2.9%) received octreotide, diazoxide, hydrocortisone, and diazepam, respectively. Two patients (5.9%) were rechallenged with TMP-SMX at a lower dose and did not experience hypoglycemia. Despite continuous intravenous glucose injection, 11 patients (42.8%) had persistent hypoglycemia within 24 hours, 7 (26.9%) had it for 28-72 hours, and 2 (7.7%) had it for 24 days and 47 days, respectively. Ultimately, 30 patients (88.2%) recovered completely without neurological sequelae, and 1 (2.9%) did not report an outcome. Three patients (8.8%) died of hypoglycemia, potential multiple myeloma and other causes, respectively. Details are shown in **Table 4**.

**Table 4:** Summary of treatment and prognosis of 34 patients.

Parameter		Value
Treatment	discontinued continued dose decreased na dextrose intravenously oral sugar carbohydrates supplements glucagon octreotide diazoxide hydrocortisone diazepam low-dose re-challenge	27 (79.4%) 2 (5.9%) 3 (8.8%) 2 (5.9%) 30 (88.2%) 2 (5.9%) 1 (2.9%) 4 (11.8%) 1 (2.9%) 1 (2.9%) 1 (2.9%) 1 (2.9%) 2 (5.9%)
Duration of hypoglycemia (26)^a^	rapid 8-24h 24-48h 48-72h 24-47d	1 (3.8%) 10 (38.4%) 7 (26.9%) 6 (23.1%) 2 (7.7%)
Outcome	recovery death na	30 (88.2%) 3 (8.8%) 1 (2.9%)

na, not applicable.

a Represents the number of patients out of 34 in whom information regarding this particular parameter was provided.

## Discussion

Hypoglycemia is characterized by low plasma glucose levels and ultimately leads to the clinical syndrome of neurological hypoglycemia with numerous etiologies (37). Patients with insulinoma, paraneoplastic hypoglycemia, hyperinsulinemic hypoglycemia syndrome, alcohol, infection, hypocortisolism, liver dysfunction, malnutrition, renal insufficiency, toxins, and drugs are associated with hypoglycemia (38). A variety of medications can induce exacerbated hypoglycemia, including acetaminophen, beta-blockers, pentamidine, ACEI, and propoxyphene (39, 40). The presence of these risk factors increases the risk of hypoglycemia in patients receiving TMP-SMX (3).

In our study, TMP-SMX -induced hypoglycemia occurred primarily in patients over 60 years of age. In these patients, the median onset of hypoglycemia was seven days. Symptoms of hypoglycemia include neurogenic (autonomic) or neuroglycopenic symptoms. The clinical signs of TMP-SMX-induced hypoglycemia are mainly neurogenic hypoglycemia. In patients with TMP-SMX-induced hypoglycemia, other factors predisposing to hypoglycemia include the use of hypoglycemic drugs (e.g., beta-blockers, ACEI, acetaminophen, propoxyphene), liver dysfunction, malnutrition, and renal insufficiency. Renal insufficiency was probably the most common risk factor for TMP-SMX-induced hypoglycemia, and 74% of patients had renal insufficiency at the time of hypoglycemia in our study. Although our retrospective analysis identified risk factors for co-trimoxazole-induced hypoglycemia, the incidence of this complication could not be determined.

About 10% to 30% of trimethoprim is metabolized to the inactive form, and the remainder is excreted unchanged in the urine. Sulfamethoxazole is mainly metabolized in the liver, and about 30% is excreted unchanged in the urine. In normal renal function, the half-life of TMP-SMX is 8-15 hours, while in end-stage renal disease, the half-life can be extended to 20-50 hours (41). Therefore, the dose of TMP-XSM should be adjusted when creatinine clearance is below 30 mL/min. This implies assessing the patient’s baseline kidney and liver function before starting co-trimoxazole is crucial. Both components of TMP-SMX can significantly affect the metabolism of concomitantly administered drugs. The trimethoprim component selectively inhibits CYP2C8, while sulfamethoxazole inhibits CYP2C9 (42). Trimethoprim may increase the risk of hypoglycemia by inhibiting repaglinide liver metabolism (21). This suggests that co-trimoxazole should be used with caution in the case of concurrent oral hypoglycemic agents.

The occurrence of hypoglycemia appears to be dose-related. Three patients had no additional hypoglycemia symptoms that occurred when the dose of co-trimoxazole was adjusted according to renal function (6, 19, 32). Hypoglycemia caused by TMP-SMX may be related to sulfamethoxazole. The possible mechanism is the structural similarity between sulfamethoxazole and sulfonylureas (10, 43). Sulfamethoxazole is postulated to increase insulin secretion, a theory supported by elevated insulin and C-peptide levels in more than 79% of patients in our study.

Currently, there is no optimal management plan for TMP-SMX-induced hypoglycemia. Opinions on continuous administration of TMP-SMX are inconsistent after hypoglycemia. The discontinuation of TMP-SMX is safe and eliminates the risk of recurrent hypoglycemia. Limited data suggest that some patients may be successfully re-challenged at lower doses. TMP-SMX remains the only option when other effective alternatives for severe PCP, such as pentamidine and primaquine, are unavailable. Intravenous glucose is needed for hypoglycemia to prevent seizures, coma, and death. Octreotide, a somatostatin analog, reduces calcium influx through voltage-gated channels in beta islet cells, thus reducing pancreatic calcium-mediated insulin release. It is commonly used in the treatment of sulfonylurea overdose (44, 45). Glucagon may be used as a treatment option in hypoglycemia refractory to glucose administration. Despite appropriate treatment, symptoms persisted for more than 8 hours in 95% of patients in our analysis.

## Conclusion

Clinicians should be aware of this rare but life-threatening hypoglycemia complication of co-trimoxazole, especially in patients with multiple risk factors. Early interventions in the event of hypoglycemia during TMP-SMX treatment are essential to prevent severe adverse outcomes. Blood glucose monitoring is feasible in patients taking long-term co-trimoxazole.

## Data availability statement

The original contributions presented in the study are included in the article/supplementary material. Further inquiries can be directed to the corresponding author.

## Author contributions

LS and CW conceived of the presented idea. CW, WF, Zl and LS wrote the manuscript. All authors contributed to the article and approved the submitted version.
